# Microbial Community Structures and Important Associations Between Soil Nutrients and the Responses of Specific Taxa to Rice-Frog Cultivation

**DOI:** 10.3389/fmicb.2019.01752

**Published:** 2019-08-06

**Authors:** Xiaomei Yi, Kai Yi, Kaikai Fang, Hui Gao, Wei Dai, Linkui Cao

**Affiliations:** ^1^School of Agriculture and Biology, Shanghai Jiao Tong University, Shanghai, China; ^2^China National Cereals, Oils and Foodstuffs Corporation, Beijing, China

**Keywords:** bacteria, archaea, fungi, rice-frog cultivation, paddy rhizosphere soil, network analysis

## Abstract

Rice-frog cultivation is a traditional farming system in China and has been reintroduced as an agricultural practice in China in recent years. The microbial community in paddy rhizospheric soils has attracted much attention because many microorganisms participate in functional processes in soils. In this study, Illumina MiSeq sequencing-based techniques were used to investigate soil microbial communities and functional gene patterns across samples obtained by conventional rice cultivation (CR) and rice-frog cultivation (RF). The results showed that RF significantly affected the microbial community composition and richness, which indicated that the rhizospheric microorganisms responded to the introduction of tiger frogs into the paddy fields. Operational taxonomic units (OTUs) from *Sandaracinaceae*, *Anaerolineaceae*, *Candidatus* Nitrososphaera, *Candidatus* Nitrosotalea, *Candidatus* Nitrosoarchaeum and some unclassified OTUs from *Euryarchaeota* and *Agaricomycetes* were significantly enriched by RF. The abiotic parameters soil organic carbon (SOC), nitrate nitrogen (NO_3_^−^-N), and available phosphorus (AP) changed under RF treatment and played essential roles in establishing the soil bacterial, archaeal, and fungal compositions. Correlations between environmental factors and microbial communities were described using network analysis. SOC was strongly correlated with *Anaerolineaceae*, *Methanosaeta*, and *Scutellinia*. NO_3_^−^-N showed strong positive correlations with *Opitutus*, *Geobacter*, and *Methanosaeta.* NH_4_^+^-N was strongly positively associated with *Sideroxydans*, and TN was strongly positively correlated with *Candidatus* Nitrotoga. Compared to conventional CR, RF greatly enriched specific microbial taxa. These taxa may be involved in the decomposition of complex organic matter and the transformation of soil nutrients, thus promoting plant growth by improving nutrient cycling. The unique patterns of microbial taxonomic and functional composition in soil profiles suggested functional redundancy in these paddy soils. RF could significantly affect the bacterial, archaeal, and fungal communities though changing SOC and AP levels.

## Introduction

In most developing countries, agriculture is the main source of food, employment, income and nutrition (Saiful Islam et al., 2015). Hence, it is necessary to sustainably increase grain production, achieve food self-sufficiency and improve the well-being of small-scale farmers (Berg and Tam, 2018). This growth in food production must be achieved by reducing the use of land, water, labor and chemicals (Doss, 2010; Yuan et al., 2017). As farmers intensify production by increasing the use of chemicals, concerns about the negative effects of those practices on human health and the environment are growing. Therefore, crop management practices need to be improved to increase productivity and minimize adverse impacts on the quality of the natural resource base (Laborte et al., 2009; Berg and Tam, 2018).

Traditional agricultural systems have shown successful adaptation to different environments and high biodiversity (Lu and Li, 2006; Suh, 2014; Teng et al., 2015; Ren et al., 2018). Recognizing the ecological heritage of these traditional agricultural systems and incorporating these unique conditions into future agricultural designs could aid the development of sustainable agricultural practices. Many studies have described the ecosystems of rice-fish, rice-turtle, rice-duck, and rice-frog cultivation and have shown how these practices reduce the use of chemical fertilizers and pesticides (Li et al., 2008; Hu et al., 2016; Zhang et al., 2016; Yang et al., 2018). It was found that rice-duck, rice-fish and rice-turtle cultivation could improve soil microbial abundance and increase the number of functional microbes, but the control of pests in paddy fields by these methods was distinctly inferior to that by rice-frog cultivation (Teng et al., 2015, 2016). In addition, previous studies have indicated that traditional rice-duck, rice-fish and rice-turtle farming methods have focused on the feeding of animals (Furuno, 2001; Luo, 2016, 2018). Frogs have been used to control pests in paddy fields for a long time. The predatory behavior of frogs in paddy fields can reduce the incidence of pests, decrease the application of pesticides and contribute to biological control (Yi et al., 2018). Although rice-frog cultivation is an ancient farming system adopted by Asian farmers for many years, this innovation may have the potential to keep up with the surge in food security demand (Sha et al., 2017).

Microorganisms are core factors affecting the biological characteristics, biogeochemical processes, and ecology of soils (Vigdis and Lise, 2002). Recent research has largely advocated that niche, biogeographic, and neutral/stochastic processes interactively determine the microbiome community composition across spatiotemporal scales in the ecosystem (Saleem et al., 2015). Soil scientists have long noticed that the natural properties of soil, such as pH, texture, and base saturation, determined by parent materials, maintain the biodiversity of soils in nature and greatly affect the basic fertility and productivity of soils during soil formation (Bai et al., 2017). In addition, anthropogenic activities such as tillage, fertilization, irrigation, and tillage have a great impact on the structure and functional properties of soil microbial communities by changing soil properties (Tripathi et al., 2015). Many recent studies have shown that the large-scale diversity and community compositions of soil microorganisms are largely driven by soil pH and some other soil properties, such as organic matter content and salinity (Su et al., 2015; Chen et al., 2016). Certain microbial taxa at high taxonomic levels can exhibit properties of ecological coherence because these taxa respond predictably to environmental variables (Li et al., 2017). Fierer et al. (2012) proposed that certain microbial phyla could be differentiated into ecologically relevant nutritional and barren categories based on substrate preferences and life strategies. Furthermore, these taxa have potential beneficial or adverse effects on crop productivity and even the stability of agro-ecosystems (Francioli et al., 2016). Under the background of exogenous organic decomposition and soil nutrient transformation, there are complex interactions between microbial taxa (Banerjee et al., 2016). Network analysis of taxon co-occurrence, as measured based on correlations between the abundances of microbial taxa, can help decipher complex microbial association patterns and the ecological rules that guide community assembly (Barberan et al., 2012). Network analysis not only reveals intertaxon associations in shared common niche spaces but also links microbial taxa to environmental parameters (Fuhrman, 2009; Ma et al., 2018). Rice frogs can directly change soil properties to influence the abundances of certain microbial phyla (Yi et al., 2018). However, knowledge regarding the changes in soil bacterial, archaeal and fungal taxa at low taxonomic levels (e.g., species, genus) in response to the introduction of tiger frogs into paddy fields remains insufficient, and network analysis of soil metagenomic-related patterns has not been done before.

We hypothesized that rice-frog cultivation systems, especially with high primary production, should be suitable for the growth of bacteria, archaea, and fungi, thus changing the microbial community structures. To test this hypothesis, rhizospheric soils were sampled from a trial field that had been subjected to rice-frog cultivation for 8 years, and Illumina MiSeq sequencing targeting the V4–V5 region of the bacterial 16S rRNA genes, 524F-10-extF/Arch958R of the archaeal 16S rRNA genes, and the fungal SSU 18S rRNA genes were used for identification and quantification of rhizospheric soil bacterial, archaeal and fungal taxa. We used network analysis to explore the potential effects of specific bacterial taxa on the relationship between microorganisms and soil nutrients.

## Materials and Methods

### Experimental Sites

The experimental base was established in the Qingpu Modern Agricultural Park of Qingpu, Shanghai (121.12° E, 31.15° N) in 2009. The experimental site for rice cultivation patterns is located in the Yangtze River Delta region. Rice is the main crop in this area, which is planted once a year. This region is a subtropical monsoon climate. The mean annual air temperature is 15.5°C, and the mean annual precipitation is 1200 mm. The soil pH of this site is approximately 6.8. Before transplantation of rice plants, the soil pH, electrical conductivity (EC), and available N (AN), available phosphorus (AP), and available potassium (AK) levels of the site were 7.40, 0.13 mS cm^–1^, 1.70 g kg^–1^, 0.38 g kg^–1^, and 0.55 g kg^–1^, respectively.

### Experimental Design

Two rice cultivation treatments were established in the experiment: conventional rice cultivation without frogs (CR) and rice-frog cultivation (RF). In both the CR and RF fields, the same amount (300 kg N ha^–1^) of nitrogen fertilizer was applied for each treatment. A randomized complete block experimental design was used with three replicates. A total of 12,000 frogs/ha were introduced into the RF paddy fields. Tiger frogs (*Rana tigrina rugulosa*), which are highly adaptable to the environment, were introduced and raised by the Zizaiyuan Agricultural Development Co., Ltd., Shanghai. At 15 days after rice transplantation, frogs large enough (≥20 g) to prey on pests were released into the paddy fields. In addition, the tiger frogs used in this study were managed in accordance with relevant guidelines and regulations of the Guide for the Care and Use of Laboratory Animals of the Ministry of Health, China. We domesticated and bred the tiger frogs with the permission of the Shanghai Forestry Bureau, and the license permission number was (2008) 419.

### Sampling and Measurements

In October 2016, samples were taken from the rhizospheric soil at the rice maturation stage. Five rice plants (with roots) were randomly selected from each plot, collected by an investigator wearing disposable gloves and then pooled. All the rhizospheric soil samples were gently scraped from the roots, and the samples were placed into aseptic sealed plastic bags and transported back to the laboratory in an ice box containing liquid N. Each soil sample was divided into two parts. One part was freeze-dried and stored at −80°C for DNA extraction. Another part was air-dried, ground and passed through a 2 mm sieve to obtain suitable powder for the analysis of soil physicochemical characteristics: soil pH, EC and AP, available potassium (AK), available N (AN), total N (TN), and soil organic carbon (SOC) levels.

### DNA Extraction

According to the manufacturer’s protocol, DNA was extracted from 0.2 g of freeze-dried soil using the E.Z.N.A.^®^ Soil DNA Kit (Omega Biotek, Norcross, GA, United States). DNA quality was evaluated by 1% sodium agarose gel electrophoresis. Using a NanoDrop 2000 spectrophotometer (Thermo Fisher Scientific Inc., Wilmington, DE, United States) to determine DNA purity and concentrations, the DNA was stored at −20°C prior to amplification.

### Amplicon Library Preparation and Sequencing

Amplicon library preparation and Illumina^®^ MiSeq sequencing (Illumina, San Diego, CA, United States) were carried out by Majorbio Biopharm Technology Co., Ltd. (Shanghai, China). The 16S rRNA V4–V5 gene fragments were amplified by the primer pair 515F/907R (F: 5′-GTGCCAGCMGCCGCGG-3′ and R: 5′-CCGTCAATTCMTTTRAGTTT-3′) (Edwards et al., 2015). For the archaeal 16S rRNA gene fragments, the primer pair used was 524F/958R (F: 5′-TGYCAGCCGCCGCGGTAA-3′ and R: 5′-YCCGGCGTTGAMTCCAATT-3′) (Besaury et al., 2014). Amplification of fungal 18S rRNA gene fragments was performed by using the forward primers SSU0817F/1196R (5′-TTAGCATGGAATAATRRAATAGGA-3′ and 5′-TCTGGACCTGGTGAGTTTCC-3′) (Xiao et al., 2018; Yuan et al., 2018). The following procedures were used for PCR: denaturation for 3 min at 95°C; 27 cycles of 30 s at 95°C, annealing for 30 s at 55°C, and elongation for 45 s at 72°C; and a final extension step for 10 min at 72°C. The PCR buffers were performed in triplicate in 20 μL mixtures containing 0.8 μL of each primer (5 μM), 2 μL of 2.5 mM dNTPs, 4 μL of 5× FastPfu buffer, 0.4 μL of FastPfu polymerase (TransGen, Beijing, China) and 10 ng of template DNA. The PCR products were extracted from 2% agarose gels, purified using the AxyPrep DNA Gel Extraction Kit (Axygen Biosciences, Union City, CA, United States) and quantified using QuantiFluor^TM^-ST (Promega, United States) according to the manufacturer’s protocol. The purified amplicons were pooled in equimolar concentrations and processed with MiSeq Reagent Kit V2, and then 250 bp paired-end dual index sequencing was performed with an Illumina MiSeq instrument (Illumina, San Diego, CA, United States). The original raw sequencing reads were submitted to the short read archives of the National Biotechnology Information Center under registration number PRJNA47103.

### Bioinformatic and Statistical Analysis of the Community

The original raw fastq files were demultiplexed, quality-filtered by Trimmomatic and merged using FLASH with the following criteria: (i) the reads were truncated at any site that received an average quality score < 20 over a 50 bp sliding window; (ii) the primers matched perfectly, allowing 2 nucleotide mismatches, and reads containing ambiguous bases were deleted; and (iii) sequences that overlapped by more than 10 bp were merged based on the overlap sequence (Magoc and Salzberg, 2011). Operational taxonomic units (OTUs) were clustered by similarity using Mothur version 1.31.1 with 97% cutoff points, and chimeric sequences were removed by quantitative insights into microbial ecology (QIIME) (Schloss et al., 2009; Caporaso et al., 2010). The taxonomy of each gene sequence was examined against the Silva (SSU123) database with a confidence threshold of 70%. A total of 298,045 high-quality 16S rRNA reads, 291,954 high-quality arch-16S rRNA reads and 300,714 high-quality 18S rRNA reads were obtained.

Statistical analyses were carried out using Statistical Product and Service Solutions (SPSS) 18.0 software and the R vegan package^1^. The remaining sequences of all the samples were rarefied to the same sequencing depth. Principal coordinates analysis (PCoA) of “the Bray-Curtis distances” was performed using the R package “pcoa.” Venn diagrams were generated with the “vennerable” package in R. Redundancy analysis (RDA) of multiple correlation variations among environmental variables (SOC, TN, AP, pH and community composition at the phylum level) was carried out by using the "rda" function, and the environmental factors were fitted with the ordination plots using the vegan package in R with 999 permutations. The differential OTU abundances were calculated by using the R package “DESeq2.” Differential abundance analysis was performed by fitting the generalized linear model with a negative binomial distribution to the normalized value of each OTU and using a Wald test to test the differential abundance. Enriched and depleted OTUs were defined as OTUs with absolute differential abundance >1.0 and *p* < 0.05.

### Network Analysis

Network analysis was conducted on microbial OTUs and soil properties (AN, NH_4_^+^-N, NO_3_^−^-N, TN, and SOC) by Pearson correlation analysis. To decrease pairwise comparisons and minimize network complexity, 200 OTUs with the highest abundances from CR and RF (adjusted *P* < 0.01) were used for network analysis. The OTUs with strongly positive (*r* > 0.8) and strongly negative (*r* < −0.8) values were used to calculate the network in Cytoscape v.3.6.1 (Shannon et al., 2003). The NetworkAnalyzer tool in Cytoscape was used to calculate the network topology characteristics.

## Results

### Soil Biochemical Properties

The soil pH, TN content and NH_4_^+^-N content showed no significant differences between treatments. RF significantly decreased the NO_3_^−^-N, AP, and AK content by 37.47, 54.30, and 28.41%, respectively, and significantly increased the SOC and AN content by 4.68 and 20.22%, respectively (Table 1).

**TABLE 1:** Soil biochemical properties between CR and RF.

	**pH**	**SOC (mg g**^–^**^1^)**	**TN (mg g**^–^**^1^)**	**NH_4_^+^-N (mg g**^–^**^1^)**	**NO_3_**^–^**-N (mg g**^–^**^1^)**	**AP (μg g**^–^**^1^)**	**AK (μg g**^–^**^1^)**	**AN (mg g**^–^**^1^)**
CR	6.77 ± 0.06a	17.30 ± 0.10b	1.80 ± 0.10a	4.95 ± 0.29a	4.11 ± 0.12a	18.58 ± 0.16a	127.00 ± 0.90a	150.43 ± 4.19b
RF	6.90 ± 0.10a	18.15 ± 0.05a	1.83 ± 0.06a	5.62 ± 0.09a	2.57 ± 0.06b	8.49 ± 1.04b	90.92 ± 2.38b	180.84 ± 7.75a

**Means ± standard deviations (n = 3). Significant differences between means at the α = 0.05 level detected by Tukey’s honestly significant difference (HSD) test are labeled with different letters. SOC, soil organic C; TN, total N; AP, available P; AK, available K; CR, conventional rice cultivation; RF, rice-frog cultivation.**

### Within-Habitat Diversity and Relative Abundances of Major Phyla

**FIGURE 1:**
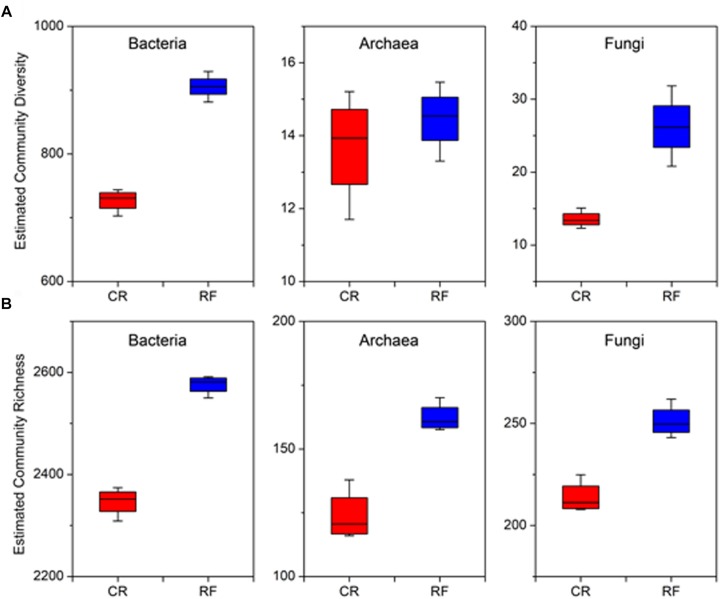
Within-sample (α-diversity) measurements between CR and RF. **(A)** Estimated species richness was calculated as *e*^*Shannon_entropy*^. **(B)** Abundance-based coverage estimator (ACE) index. The horizontal bars within the boxes represent the median. The tops and bottoms of the boxes represent the 75th and 25th quartiles, respectively. The upper and lower whiskers extend 1.5× the interquartile range from the upper and lower edges of each box, respectively. All outliers are plotted as individual points. CR, conventional rice cultivation; RF, rice-frog cultivation.

**FIGURE 2:**
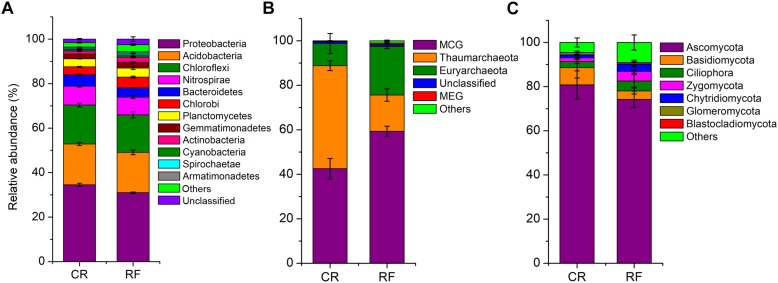
Histograms of the relative abundances of microbial phyla between CR and RF. **(A)** Relative abundances of bacterial phyla. **(B)** Relative abundances of archaeal phyla. **(C)** Relative abundances of fungal phyla. CR, conventional rice cultivation; RF, rice-frog cultivation.

Measurement of α-diversity revealed differences in community diversity between RF and CR. We calculated the abundance-based coverage estimator (ACE) index to estimate the richness of each soil sample. The Shannon diversity index was used to evaluate the microbial diversity of each soil sample. The mean estimated community diversity was higher in the RF rhizospheric soil than in the CR soil, however, there was no statistically significant difference in the archaeal community between these two treatments (Figure 1A). Compared with the CR treatment, RF significantly increased the ACE richness indexes of the bacterial, archaeal and fungal communities (Figure 1B). There were significant differences in the proportions of various phyla across the treatments. *Proteobacteria* (31.01–34.52%), *Acidobacteria* (18.08–18.34%), and *Chloroflexi* (16.82–17.51%) were the dominant bacterial phyla across treatments (Figure 2A); MCG (*Miscellaneous Crenarchaeotal Group*, 42.52–59.34%), *Thaumarchaeota* (16.31–46.32%), and *Euryarchaeota* (9.92–21.84%) were the dominant archaeal phyla (Figure 2B); and *Ascomycota* (74.2–59.34%) and *Basidiomycota* (3.83–7.81%) were the dominant fungal phyla (Figure 2C). The RF soil had a notably greater proportion of MCG and *Euryarchaeota* than the CR rhizospheric soil, whereas *Proteobacteria*, *Acidobacteria*, *Thaumarchaeota*, *Ascomycota*, and *Basidiomycota* were mostly depleted in the RF treatment.

### OTUs Enriched and Depleted by Rice-Frog Cultivation

**FIGURE 3:**
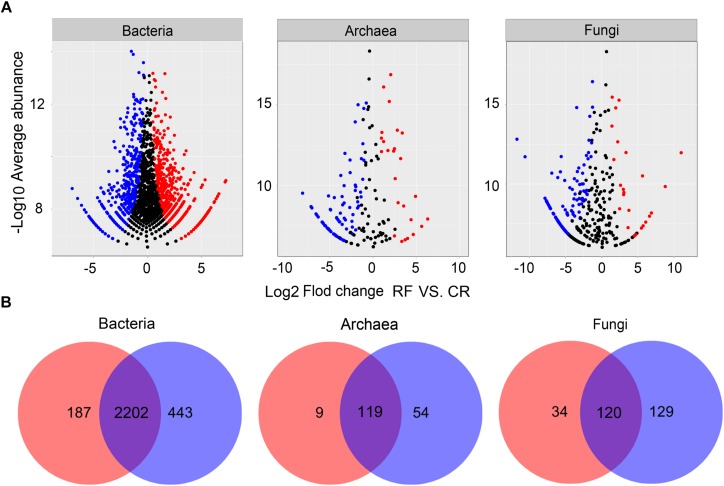
**(A)** Volcano plots illustrating OTUs that were significantly enriched (red) and depleted (blue) by RF compared with CR as determined by differential abundance analysis. Each point represents an individual OTU, and the Y-axis indicates the fold change in abundance. **(B)** Number of differential OTUs between each treatment. CR, conventional rice cultivation; RF, rice-frog cultivation.

We performed differential abundance analysis to confirm OTUs that were greatly influenced by RF (Figure 3A). Using OTU abundances from CR as a control and no more than 0.05 as an adjusted *p-*value cutoff, “enriched OTUs (eOTUs)” and “depleted OTUs (dOTUs)” were identified, representing OTUs for which the relative abundance significantly increased and decreased, respectively, by more than twofold in response to RF. There were 483, 25, and 30 eOTUs and 645, 88, and 115 dOTUs in bacteria, archaea and fungi, respectively. Among the top 10 most influential OTUs in bacteria, most eOTUs were identified as a number of unclassified members of *Methylophilaceae, Sandaracinaceae*, and *Anaerolineaceae.* Among the top 10 most influential OTUs in archaea and fungi, the eOTUs were chiefly identified as *Candidatus* Nitrososphaera, *Candidatus* Nitrosotalea, *Candidatus* Nitrosoarchaeum, some unclassified OTUs of *Euryarchaeota*, several unclassified members of *Agaricomycetes*, and *Incertae Sedis* of *Zygomycota* and *Glomeromycota.* The Venn plot (Figure 3B) showed that the bacterial, archaeal, and fungal communities shared 2202, 119, and 120 OTUs, respectively.

### Community Structure, Variation, and Determinants

**FIGURE 4:**
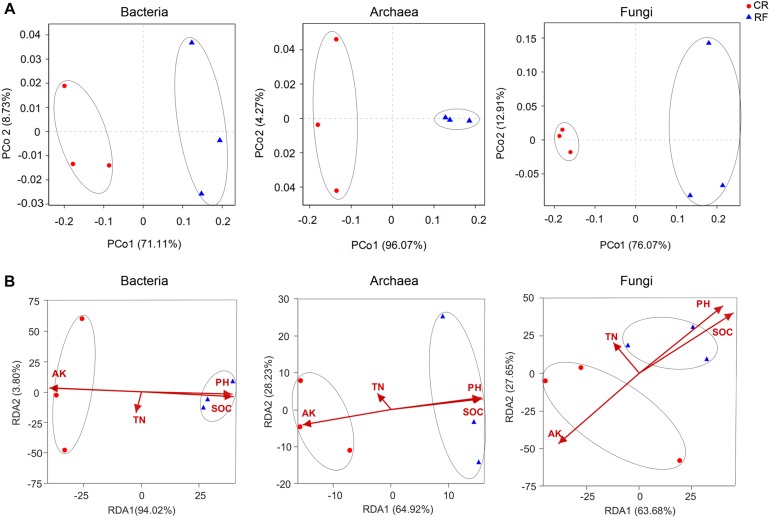
**(A)** Principal coordinates analysis (PCoA) of bacterial, archaeal, and fungal community variations between CR (conventional rice cultivation) and RF (rice-frog cultivation). **(B)** Redundancy analysis (RDA) based on Bray-Curtis dissimilarities of bacterial, archaeal and fungal communities and soil properties. AP, available phosphorus; TN, total nitrogen; pH, hydrogen ion concentration; SOC, soil organic C.

PCoA with Bray-Curtis distances showed that the community distinctly separated RF from CR along the first principal coordinate (Figure 4A). This result indicated that RF had a certain influence on bacterial, archaeal and fungal communities. RDA was used to quantify the influences of edaphic factors (i.e., pH, SOC, TN, and AP) on the bacterial, archaeal and fungal community compositions. The four constrained factors considerably contributed to the bacterial community (*P* = 0.03), archaeal community (*P* = 0.04), and fungal community (*P* = 0.03). SOC was the determinant of these factors in the bacterial community, however, AP was the determinant in the archaeal and fungal communities (Figure 4B). These results indicated that the soil bacterial community composition was mostly driven by the SOC and AP levels under long-term fertilization.

### Network Associations Among OTUs and Soil Properties

**FIGURE 5:**
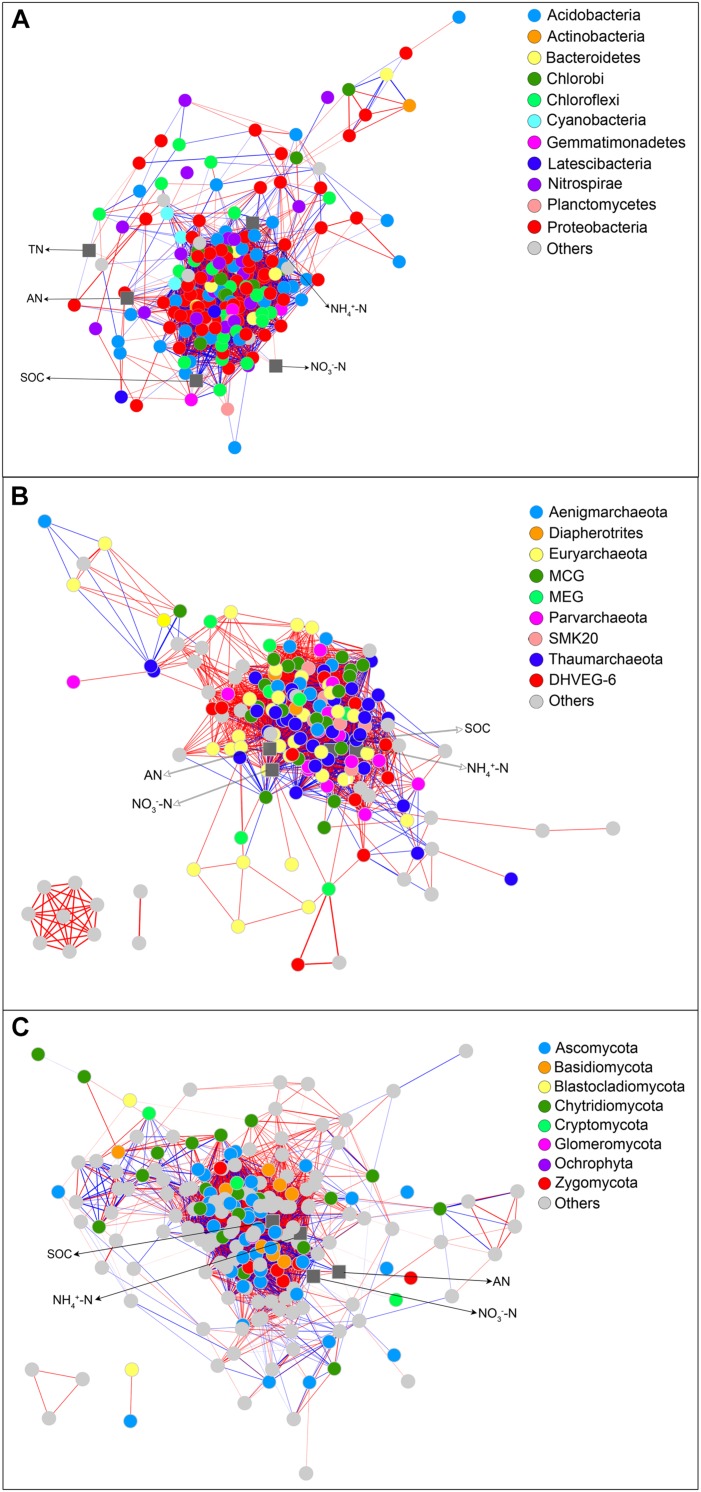
Correlation network between differentially abundant OTUs in bacterial **(A)**, archaeal **(B)**, and fungal **(C)** communities and soil properties. Blue and red lines represent significantly negative (*P* < 0.01, *r* < −0.8) and positive (*P* < 0.01, *r* > 0.8) correlations, respectively. Colored nodes signify corresponding OTUs assigned to major phyla. Soil properties are indicated with a rounded rectangle. SOC, soil organic C; TN, total N; NO_3_^−^-N, nitrate nitrogen; NH_4_^+^-N, ammonium nitrogen. CR, conventional rice cultivation; RF, rice-frog cultivation.

The bacterial network consisted of 3920 significant associations (edges) of 202 nodes, with an average clustering coefficient of 0.56 and a total diameter of 38 edges. The network showed an average number of 44 neighbors and a feature path length of 2.43. The network edges were predominantly composed of strong positive associations, and the dominant identifiable OTUs belonged to *Proteobacteria*, *Acidobacteria*, and *Nitrospirae* (Figure 5A). AN showed strong positive associations with one *Chloroflexi* KD4-96 member and *Acidobacteria* subgroup 6, subgroup 17, and subgroup 18 members and a strong negative association with *Geobacter*. NH_4_^+^-N showed strong associations with *Sideroxydans*, *Methylosarcina*, etc., and strong negative associations with *Acidibacter, Nitrospira*, etc. NO_3_^−^-N was positively correlated with *Opitutus*, *Geobacter*, etc., and negatively correlated with *Planctomyces*, *Geobacter*, etc. SOC showed a strong positive association with one uncultured member belonging to *Anaerolineaceae* and negative associations with *Planctomyces*, *Geobacter*, etc. TN was positively correlated with *Candidatus* Nitrotoga and one member of *Syntrophobacteraceae*. The archaeal network included 3116 notable associations (edges) of 178 nodes, with an average clustering coefficient of 0.63 and a total diameter of 35 edges. The network showed an average number of neighbors of 39 and a typical path length of 2.31. The network edges were significantly composed of strongly positive correlations, and the identifiable OTUs mainly belonged to the phyla *Thaumarchaeota* and *Euryarchaeota* (Figure 5B). AN showed strong positive associations with *MCG* members and *Methanosaeta*, etc. NH_4_^+^-N was positively correlated with *Methanobacterium*, etc. NO_3_^−^-N showed a strong negative association with *Methanosaeta*, etc. SOC was positively correlated with one member within Rice Cluster II and *Methanosaeta*, etc. and negatively correlated with *Candidatus* Nitrosoarchaeum, etc. The fungal network consisted of 3464 significant associations (edges) of 208 nodes, with an average clustering coefficient of 0.57 and a total diameter of 33 edges. The fungal network showed 38 neighbors and a typical path length of 2.51. The network edges predominantly consisted of strongly positive and negative correlations, and the dominant identifiable OTUs belonged to *Ascomycota*, Chytridiomycota and *Euryarchaeota* (Figure 5C). AN showed strong positive associations with *Incertae Sedis* within *Zygomycota* and a negative association with *Incertae Sedis* within *Glomeromycota*. NH_4_^+^-N was strongly positively associated with *Cochliobolus*, etc., and negatively associated with *Trimastix*, etc. NO_3_^−^-N showed a strong association with *Incertae Sedis* within *Ascomycota*, etc., SOC was strongly positively correlated with *Scutellinia*, etc.

## Discussion

### Changes in Microbial Community Composition in Response to Rice-Frog Cultivation

To the best of our knowledge, this study was the first comprehensive investigation of the structural and functional differentiation of microbial communities in rice paddy soil under rice-frog cultivation. RF treatment was used as an organic fertilizer and could be considered an organic treatment. The observed Shannon index showed that the RF treatment significantly increased the microbial community diversity compared to that of the CR treatment. The bacterial, archaeal and fungal community richness was significantly enhanced by RF. Similar results have been observed in previous studies for paddy soil (Zhong et al., 2009; Geisseler and Scow, 2014; Wang et al., 2017). RF had an important influence on the structure and function of the rice rhizospheric soil. The phyla *Proteobacteria*, *Acidobacteria*, and *Chloroflexi* dominated bacterial communities across all treatments and roughly corresponded to the results of previous studies on rice rhizospheric soils (Edwards et al., 2015). Most paddy soils have abundant nutritional lifestyles; generally, the phylum *Proteobacteria* accounts for the largest proportion of the soil bacterial communities in terms of both metabolism and genetics, even in other paddy soils (Yuan et al., 2018). Phylum *Chloroflexi* is a facultative anaerobic bacterium that plays a recognized role in heterotrophic oligotrophs in soils, showing the viability of recalcitrant plant polymers (Hug et al., 2013).

RF had no significant effect on archaeal community diversity. However, the relative abundances of three dominant phyla, namely, *MCG*, *Thaumarchaeota*, and *Euryarchaeota*, changed significantly. The phylum *Thaumarchaeota* constituted the largest fraction of the soil bacterial communities in the CR rhizospheric soil, however, the relative abundance of the phylum *Thaumarchaeota* was lower than that of *MCG* or *Euryarchaeota* in RF. Euryarchaeota is the dominant phylum in a large number of soil environments (Lee et al., 2015; Liu et al., 2016). *Mesophilic Crenarchaea* have recently been considered to play a key role in soil nitrogen and carbon cycling (Lam et al., 2007; Brochier-Armanet et al., 2008). MCG species have been detected in a variety of environmental conditions, including marine and terrestrial environments, as well as oxic and anoxic geochemical zones. MCG species are heterotrophic, including ubiquitously distributed and dominant MCGs in systems with a high content of organic carbon in the sediment (Meador et al., 2015). *Euryarchaeota*, the second dominant archaeal phylum identified in our study, plays notable roles in anaerobic degradation through nitrate reduction (Cabello et al., 2004), nitrogen fixation (Raymond et al., 2004), organic matter degradation (Ke et al., 2014), methane oxidation (Walter et al., 2002), and the metabolism of sulfur and iron (Edwards, 2000). These species exist in all major habitats of the rice field ecosystem, especially those belonging to the classes *Methanobacteria* and *Methanomicrobia*, which are involved in greenhouse gas production, with CH_4_ as the final product of anaerobic respiration (Hu et al., 2013).

Fungi, as an important component of microbial communities, promote soil organic matter cycling, nutrient transformation, toxic degradation, and crop diseases. Fungal community diversity is a prerequisite for the maintenance of the normal functions of soil ecosystems (Liu et al., 2016). Similar to most other flooded paddy soils (Yuan et al., 2018), the dominant fungi in our samples were members of the phylum *Ascomycota*, which is consistent with our hypothesis. RF significantly increased the fungal diversity and the relative abundance of *Zygomycota* but decreased the relative abundances of *Ascomycota* and *Basidiomycota.* Based on the results, we can conclude that although *Ascomycota* was the dominant fungal phylum, the abundance of this phylum was significantly reduced in RF. The frogs introduced into RF produced frog dung, which increased the nutrient content and the fungal abundance. The differences in nutrition, including different quantities of frogs and fertilizers, may also affect the diversity and richness of fungi (Thormann, 2006; Broeckling et al., 2008). Higher species diversity and nutrition reduced the abundance of pathogenic fungi (Wang et al., 2017), which may have significantly decreased the relative abundance of *Ascomycota* in RF.

### Rice Frogs Influenced the Taxonomic Abundances in the Microbial Community

We performed differential abundance analysis to distinguish OTUs that were responsible for the observed microbial community differences between the RF and CR rhizospheric soils. The OTUs from the phyla *Betaproteobacteria*, *Gammaproteobacteria*, *Chloroflexi*, and *Acidobacteria* were the most significantly enriched by RF. Chen et al. (2015) declared that the phyla *Betaproteobacteria*, *Gammaproteobacteria*, and *Chloroflexi* thrive under conditions with high substrate availability. Although there are many oligotrophic members in the phylum *Acidobacteria* (Pascault et al., 2013), some members of *Acidobacteria* were depleted by RF, while others were largely enriched. Our results were consistent with previous studies that reported that several members of *Acidobacteria* (e.g., subgroups 1 and 7) exhibited very low abundance, while others (e.g., subgroups 4 and 6) were extremely abundant in soils with rich SOC content (Liu et al., 2014). We analyzed the top 10 most OTUs that were influenced at the genus level and observed that the OTUs that were most highly enriched by RF were several unclassified members of the Methylop*hilaceae*, *Anaerolineaceae*, and *Sandaracinaceae* families. Soluble simple organic matter can provide electronic donors for aerobic denitrification of *Methylophilaceae* of *Betaproteobacteria* under microaerobic conditions, and aerobic denitrification can be performed by using nitrate or nitrite (Cao et al., 2019). *Anaerolineaceae* species are keystone microbes that participate in the degradation of organic matter (Meng et al., 2019). The species of *Sandaracinaceae* are heterotrophic consumers of low-molecular-weight organic compounds, such as ethanol, hydrogen, butyrate, and acetate (Probandt et al., 2017). In our study, the specific bacterial taxa substantially enriched by RF play indispensable roles in organic decomposition and soil C, N, and P transformation.

For the archaeal community, the OTUs from *Thaumarchaeota* were the most significantly enriched by RF. At the genus level, the main enriched OTUs in the rhizospheric soil were *Candidatus* Nitrososphaera, *Candidatus* Nitrosotalea, *Candidatus* Nitrosoarchaeum and several members of SAGMCG-1 (South African Gold Mine Group 1). *Candidatus Nitrososphaera*, *Candidatus Nitrosotalea*, and *Candidatus Nitrosoarchaeum* all belong to ammonia-oxidizing archaea. Ammonia oxidation is the first and rate-limiting step of nitrification and is dominated by ammonia-oxidizing archaea (AOA) and ammonia-oxidizing bacteria (AOB) (Fitzgerald et al., 2015). The abundance of AOA, which dominate soil microbial activity, is usually greater than that of AOB (Lehtovirta-Morley et al., 2016). Previous studies showed that AOA had the capacity to grow mixotrophically by assimilating organics (Hallam et al., 2006; Walker et al., 2010; Liu et al., 2018). A recent study showed that some organics (pyruvate, oxaloacetate) could detoxify intracellular H_2_O_2_, functioning as chemical scavengers rather than archaeal membrane lipids, indicating that they have strict autotrophic growth (Kim et al., 2016). These results may explain why the relative abundance of AOA increased in RF.

In terms of fungi, the OTUs from *Glomeromycota*, *Basidiomycota*, and *Zygomycota* were the most significantly enriched by RF. These results were consistent with those of previous studies (Lynch and Thorn, 2006; Wang et al., 2017). At the class level, the main enriched OTU in the rhizospheric soil was *Agaricomycetes* after RF. Most *Agaricomycetes* species are saprotrophic species and play key roles in the decomposition of organic matter, such as wood and plant litter (Hibbett, 2007; Wang J. et al., 2018; Wang M.M. et al., 2018), which might explain the high relative abundance of *Agaricomycetes* in RF. Furthermore, some members of *Agaricomycetes* are known to be ectomycorrhizal fungi that can mobilize nutrients from organic substrates (Tibbett, 2002) and are conducive to plant growth. In addition, RF could greatly increase the relative abundance of the phylum *Zygomycota*.

### Correlations Between Environmental Factors and the Microbial Community

PCA plots revealed that all the bacterial, archaeal and fungal communities were significantly changed by introducing tiger frogs into the rice paddy, which is roughly consistent with previous studies (Liu et al., 2017; Wang et al., 2017). Further Mantel test analysis revealed that soil AP was significantly correlated with both archaeal and fungal community compositions in the soil. However, SOC was significantly correlated with the bacterial community. SOC and AP levels have been previously identified as key factors that influence microbial community composition (Dong et al., 2014; Liu et al., 2017).

C and N are essential resources for microbial growth (Li et al., 2017). Soil C and N levels are closely associated with specific taxa that are significantly affected by RF. Our hypothesis was confirmed by a network analysis based on co-occurrence, which revealed strong positive correlations of SOC with some taxa, e.g., one uncultured member belonging to *Anaerolineaceae* from *Chloroflexi*, Rice Cluster II and *Methanosaeta* from *Euryarchaeota*, and *Scutellinia* from *Ascomycota*. NO_3_^−^-N showed strong positive correlations with *Opitutus*, *Geobacter*, and *Methanosaeta.* NH_4_^+^-N was strongly positively associated with *Sideroxydans* and *Methylosarcina* from *Proteobacteria*, *Methanobacterium* from *Euryarchaeota*, and *Cochliobolus* from *Ascomycota*. TN was strongly positively correlated with *Candidatus* Nitrotoga from *Proteobacteria*. Studies have indicated that *Anaerolineaceae* species are keystone microbes that participate in degrading organic matter (In’t Zandt et al., 2018; Meng et al., 2019). Previously, *Methanosaeta* species have been believed to be limited to acetate, which is a substrate for methane production, living in wastewater, marshes and wetlands and releasing methane and carbon dioxide by breaking down the acetic salts produced by other microbes in the environment (Barua et al., 2018; Ji et al., 2018). In addition, as the dominant bacteria in the aggregates, *Geobacter* species were highly connected with NO_3_^−^-N and expressed genes related to ethanol metabolism, suggesting that *Geobacter* and *Methanosaeta* species exchange electrons through direct interspecific electron transfer (Rotaru et al., 2014). Cocultures of *Geobacter* and *Methanosaeta* stoichiometrically convert ethanol to methane. As a genus of cup fungi, *Scutellinia* belongs to the family *Pyronemataceae*. The strong correlation between this genus and plant growth indicates possible beneficial effects of *Scutellinia* in reducing plant disease in soils (Franke-Whittle et al., 2015). *Opitutus* species are dominant denitrifying bacteria (Tanikawa et al., 2018), so the abundance of *Opitutus* species was strongly associated with the NO_3_^−^-N content. *Sideroxydans* is a genus of Fe-oxidizing bacteria that is associated with iron cycling in freshwater and marine environments and groundwater, as well as in most sediments and soils (Emerson et al., 2010). Nitrification is an important process in biogeochemical N-cycling and biological wastewater treatment. The second step, oxidation of nitrite to nitrate, is catalyzed by nitrite-oxidizing bacteria (NOB). Uncultured NOB of the genus *Candidatus* Nitrotoga are widely distributed in both natural and engineered ecosystems (Kitzinger et al., 2018). *Candidatus* Nitrotoga species exhibit metabolic activity under low-oxygen or anoxic conditions, which expands the environmental niche for *Candidatus* Nitrotoga, similar to that of other NOB (Boddicker and Mosier, 2018). Our results showing that the relative abundance of *Candidatus* Nitrotoga was positively associated with the TN content were roughly consistent with previous conclusions.

## Conclusion

In the present study, 8 years RF significantly affected the microbial composition and structure. OTUs from *Sandaracinaceae*, *Anaerolineaceae*, *Candidatus* Nitrososphaera, *Candidatus* Nitrosotalea, and *Candidatus* Nitrosoarchaeum and some unclassified OTUs of *Euryarchaeota* and *Agaricomycetes* were significantly enriched by RF. The abiotic parameters SOC, NO_3_^−^-N, and AP changed in the RF treatment, which played important roles in the soil bacterial, archaeal, and fungal compositions. Furthermore, correlations between environmental factors and microbial communities were described using network analysis. SOC was strongly correlated with *Anaerolineaceae*, *Methanosaeta*, and *Scutellinia*. NO_3_^−^-N showed strong positive correlations with *Opitutus*, *Geobacter*, and *Methanosaeta.* NH_4_^+^-N was strongly positively associated with *Sideroxydans*, and TN was strongly positively correlated with *Candidatus* Nitrotoga. Compared to conventional rice cultivation, RF greatly enriched specific microbial taxa. These taxa are involved in the degradation of complex organic matter and the conversion of soil nutrients, thus improving nutrient utilization to promote plant growth.

## Data Availability

The datasets generated for this study can be found in NCBI, PRJNA47103.

## Author Contributions

XY and LC contributed to the conception and design of the study, acquisition, analysis, interpretation of data, drafting, revising, and final approval of the manuscript. KY, KF, HG, and WD contributed to the editing and final approval of the manuscript.

## Conflict of Interest Statement

The authors declare that the research was conducted in the absence of any commercial or financial relationships that could be construed as a potential conflict of interest.

## Footnotes

1 http://www.r-project.org
