# Erratum: Microbial Community Structures and Important Associations Between Soil Nutrients and the Responses of Specific Taxa to Rice-Frog Cultivation

**DOI:** 10.3389/fmicb.2019.02206

**Published:** 2019-09-24

**Authors:** Frontiers Production Office

**Affiliations:** Frontiers Media SA, Lausanne, Switzerland

**Keywords:** bacteria, archaea, fungi, rice-frog cultivation, paddy rhizosphere soil, network analysis

Due to a typesetting error, there was a mistake in the ***Abstract***. Instead of “${\text{NH}}_{4}^{++}$-N” it should be “${\text{NH}}_{4}^{+}$-N” which was strongly positively associated with *Sideroxydans*.

The publisher apologizes for the error. The original article has been updated.

